# A Radiosonde Using a Humidity Sensor Array with a Platinum Resistance Heater and Multi-Sensor Data Fusion

**DOI:** 10.3390/s130708977

**Published:** 2013-07-12

**Authors:** Yunbo Shi, Yi Luo, Wenjie Zhao, Chunxue Shang, Yadong Wang, Yinsheng Chen

**Affiliations:** 1 The Higher Educational Key Laboratory for Measuring & Control Technology and Instrumentations of Heilongjiang Province, School of Measurement-Control Technology & Communications Engineering, Harbin University of Science and Technology, Harbin 150080, China; E-Mails: shiyunbo@hrbust.edu.cn (Y.L.); zwjsky888@163.com (W.Z.); scxlcxy@163.com (C.S.); wydhust@163.com (Y.W.); 2 Harbin Institute of Technology, Harbin 150001, China; E-Mail: chen_yinsheng@126.com

**Keywords:** radiosonde, CPU, temperature sensor, humidity sensor, MEMS, sensor array, condensation, multi-sensor data fusion

## Abstract

This paper describes the design and implementation of a radiosonde which can measure the meteorological temperature, humidity, pressure, and other atmospheric data. The system is composed of a CPU, microwave module, temperature sensor, pressure sensor and humidity sensor array. In order to effectively solve the humidity sensor condensation problem due to the low temperatures in the high altitude environment, a capacitive humidity sensor including four humidity sensors to collect meteorological humidity and a platinum resistance heater was developed using micro-electro-mechanical-system (MEMS) technology. A platinum resistance wire with 99.999% purity and 0.023 mm in diameter was used to obtain the meteorological temperature. A multi-sensor data fusion technique was applied to process the atmospheric data. Static and dynamic experimental results show that the designed humidity sensor with platinum resistance heater can effectively tackle the sensor condensation problem, shorten response times and enhance sensitivity. The humidity sensor array can improve measurement accuracy and obtain a reliable initial meteorological humidity data, while the multi-sensor data fusion technique eliminates the uncertainty in the measurement. The radiosonde can accurately reflect the meteorological changes.

## 1. Introduction

The development of radiosonde systems can be traced back to the 1930s. In 1928, in the former Soviet Union Moerqiafu invented the radiosonde, which had the advantages of compact volume, simple observation technique, and the reliable detection results. Its measurement range was from 10 to 15 km. and soon it was widely used to acquire meteorological data. Vaisala, who is the founder of the Vaisala Company, invented the first electromechanical radiosonde. In the 1980s, the production of the analog electronic radiosonde RS80 ocurred, and in recent years, a digitized RS92 radiosonde was developed [1]. The radiosonde can provide long-term and high-quality climate records, calibrate and test the measurement instruments, including satellites and other remote sensing data quality, and provide a greater range of atmospheric data. Radiosondes have been widely used in many fields, such as military, aerospace, weather forecasting, and so on [2]. The radiosonde can collect meteorological data from the ground to a height of 35,000 m, and consists normally of a humidity sensor, temperature sensor and pressure sensor with low temperature resistance, low power consumption and reliable performance. Additionally, to the best of the authors' knowledge there are some urgent issues to be solved solved. The first is how to further improve the response speed of humidity sensors, and the other is how to solve the failure problems caused by condensation on the humidity sensor in the harsh environment and optimize the data handling algorithms.

Meteorological changes are closely linked with people's lives, and directly affect people engaged in industry, agriculture, military, aerospace, and so on [3–7]. In the field of meteorological detection, humidity is an important parameter, so a humidity sensor needs to have good linearity, high sensitivity, fast response time, good reliability and repeatability, and offer a wide range of humidity measurements [8,9]. Currently, meteorological detection humidity sensors can be divided into two categories, the first one is the resistive humidity sensor, and the other is the capacitive humidity sensor [10,11]. The most sensitive material used in resistive humidity sensors is LiCl [12], and this type of humidity sensor has the advantages of good sensing performance and good reliability, but its time response is slow and it presents undesired limitations in low or high humidity environments [13]. The capacitive humidity sensor is being widely used thank to its high sensitivity, low power consumption, fast response time and low fabrication cost [14]. This type of humidity sensor commonly uses a polyimide material to prepare a humidity sensitive layer due to its good sensing performance [15,16]. Its humidity sensing principle is as follows: water vapor can affect the dielectric constant value, and the capacitor value of the sensor is proportional to the relative humidity [17–20], however, due to its sensitive film material has poor performance under low temperature conditions, and hence most capacitive humidity sensors have poor linearity. The humidity value needs to be processed when the temperature is below −20 °C. The method of heating the humidity sensor can solve the humidity sensor failure problems due to the sensor material disadvantages in low temperature and high humidity environment. Kang and Wise conducted related research, and proved that this method can effectively solve the humidity condensation problems and improve the sensor response speed [15].

Currently, most of radiosondes have a single humidity sensor, and the data processing method used is the simple arithmetic average method. In order to make the humidity data more reliable, eliminate the measurement uncertainty and avoid affecting the measurement results because of humidity sensor failure, multi-sensor arrays and multi-sensor data fusion technology should be applied in the meteorological detection field [21].

In this paper, a sandwich plate capacitive humidity sensor, integrated with a platinum resistance heater, is fabricated using micro electro mechanical system (MEMS) technology. The sensitive material of the sensor is polyimide. The heating power is regulated according to the environmental temperature to allow the humidity sensor to work under optimal temperature conditions. Moreover, the designed structure can solve the condensation problem of humidity sensors under the low temperature condition. The advantages of the humidity sensor are fast response time, high sensitivity and better linearity.

**Figure 1.**
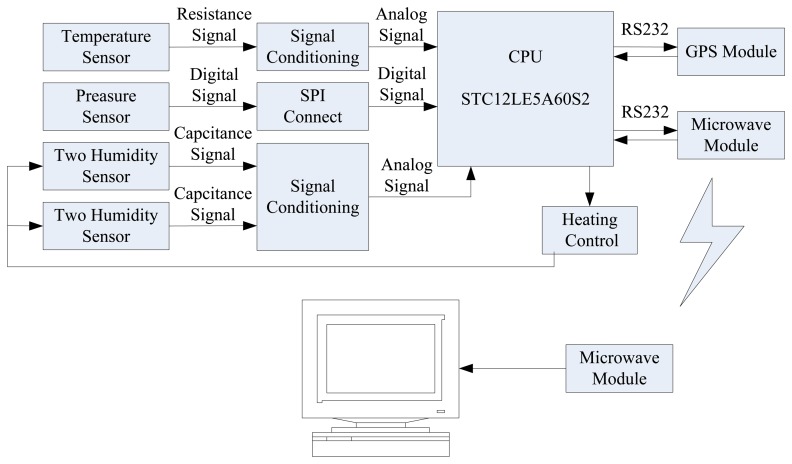
System block diagram.

In order to eliminate the uncertainty in the measurement, to further improve the consistency and reliability of humidity data, and to enhance the reliability and accuracy of the data, four humidity sensors are used to form a sensor array to acquire the humidity information. The weighted average method and a batch estimatation data fusion algorithm are used to process the initial humidity values. Figure 1 shows the system block diagram.

## 2. Multi-Sensor Data Fusion

One of the important aspects of using multi-sensor data fusion technology is to select a suitable fusion algorithm [22]. Multi-sensor data fusion can be basically summarized in two categories: random and artificial intelligence. The random class includes weighted average method, Kalman filter method Multi-Bayesian estimation method, Dempsder-Shafer (D-S) evidence reasoning, and production rule; In the artificial intelligence class there are neural networks, rough set theory, expert systems, and so on [23–26]. A variety of multi-sensor data fusion algorithms exist that are mutually complementary, so selecting two or more data fusion algorithms for data fusion is often possible to achieve better results.

Because the system needs faster processing data speed and better real-time performance, the computational complexity of data fusion technology is not suitable. Besides, the sensor array is made of four humidity sensors, and the number of the humidity sensors is small. Taking all factors into consideration, the weighted average method and batch estimate method are used to process humidity data. Detailed are described as follows: The Grubbs Criterion is used to judge and to remove invalid humidity data due to the humidity sensor failure. If one of the measured humidity data (*H_Ei_*) residual errors (*V_i_*) satisfies the following formula:

(1) $$\left|V_{i}\right|>\lambda \left(a,n\right)\sigma$$

then *H_Ei_* is a bad value, and we remove it [27]. The term *λ* stands for the Grubbs Criterion discriminant coefficient, *a* stands for degree of significance, and its value is generally 0.05 or 0.01, *n* represents the measurement time, and *σ* is the standard deviation. The four groups of humidity data, processed by the Grubbs Criterion, are divided into two groups. Each group of average values (*H*_1_, *H*_2_) and variances (*σ*_1_, *σ*_2_) are calculated. After removing the invalid humidity data, the consistent data is finally divided into two groups (*H*_1_*_m_* and *H*_2_*_m_*). The data weighted average of the two groups are calculated by:

(2) $$\bar{H_{1}}=\frac{1}{m}\sum_{i=1}^{m}p_{1i}H_{1i}$$

(3) $$\bar{H_{2}}=\frac{1}{n}\sum_{i=1}^{n}p_{2n}H_{2n}$$

where *P*_1_*_i_* stands for the weight of the first group corresponding to the respective humidity data, *P*_2_*_n_* stands for the weight of the second group corresponding to the respective humidity data. The standard deviation can be calculated by:

(4) $$\sigma_{1}=\sqrt{\frac{\sum_{i=1}^{m}{\left(H_{1i}-{\bar{H}}_{1}\right)}^{2}}{m-1}}$$

(5) $$\sigma_{2}=\sqrt{\frac{\sum_{i=1}^{m}{\left(H_{2i}-{\bar{H}}_{2}\right)}^{2}}{n-1}}$$

Finally, the expression of ideal humidity can be obtained as follows:

(6) $$H_{s}=\frac{\sigma_{2}^{2}}{\sigma_{1}^{2}+\sigma_{2}^{2}}\bar{H_{1}}+\frac{\sigma_{2}^{2}}{\sigma_{1}^{2}+\sigma_{2}^{2}}\bar{H_{2}}$$

Using the determinate coefficient (*R*) and the sum of variance (*SSE*) to evaluate the expression. *SSE* stands for the square of the error when calculating the sum of the actual humidity (*H_E_*) and theoretical humidity (*H_R_*) and its formula is as follows:

(7) $$\text{SSE}=\sum_{i=1}^{n}{\left(H_{Ei}-H_{R}\right)}^{2}$$

The closer *SSE* gets to 0, the closer the theoretical humidity value is to the actual one, indicating a more accurate prediction of humidity data. *SSR* stands for the square of the error of calculating the sum of the actual humidity (*H_E_*) and theoretical humidity mean (*H̄_R_*), and the corresponding formula is as follows:

(8) $$\text{SSR}=\sum_{i=1}^{n}{\left(H_{Ei}-{\bar{H}}_{Ri}\right)}^{2}$$

*SST* stands for sum of the actual humidity (*H_E_*) and actual humidity mean (*H̄_E_*) square of the error, the formula is as follows:

(9) $$\text{SST}=\sum_{i=1}^{n}{\left(H_{Ei}-{\bar{H}}_{Ei}\right)}^{2}$$

The *R* expression is as follows:

(10) $$R=\frac{\text{SSR}}{\text{SST}}=\frac{\text{SST}-\text{SSE}}{\text{SST}}=1-\frac{\text{SSE}}{\text{SST}}=1-\frac{\sum_{i=1}^{n}{\left(H_{Ei}-H_{R}\right)}^{2}}{\sum_{i=1}^{n}{\left(H_{Ei}-{\bar{H}}_{Ei}\right)}^{2}}$$

The value of *R* is in the range [0, 1], when *R* > 0, meaning that the theoretical humidity data and actual humidity data are related; When *R* = 1, it means the theoretical humidity data coincide with the actual humidity data; and when the *R* = 0, it means the theoretical humidity data and actual humidity data are not related.

## 3. Design and Property Measurements of the Temperature Sensor

### 3.1. Design of the Temperature Sensor

**Figure 2.**
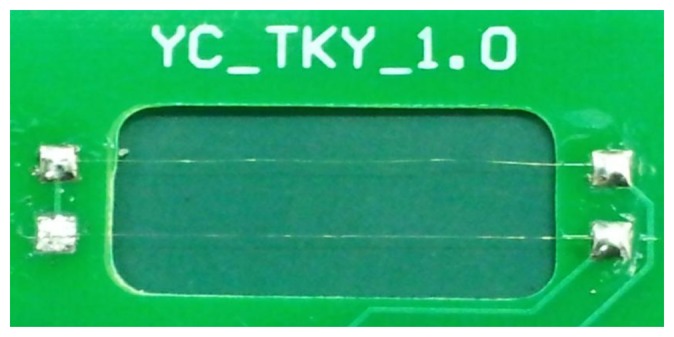
Photograph of the platinum resistance wire and temperature sensor.

The platinum resistance wire (99.999% purity), with a diameter of 0.023 mm, is used to get the atmospheric temperature aloft. A photograph of the platinum resistance wire and temperature sensor is shown in Figure 2. The resistance of the temperature sensor is about 10 Ω at room temperature.

Equations (11) and (12) are the resistance expression of platinum resistance wire under different temperature conditions.

(11) $$R_{t}=R_{0}[1+At+Bt^{2}+C\left(t-100\right)t^{2}],-200{}^{\circ}\text{C}\;<t<0{}^{\circ}\text{C}$$

(12) $$R_{t}=R_{0}(1+At+Bt^{2}),0{}^{\circ}\text{C}<850{}^{\circ}\text{C}$$

where *A* = 3.9083 × 10^−3^ °C ^−1^, *B* = −5.775 × 10^−7^ °C ^−2^, *C* = −4.183 × 10^−12^ °C ^−3^, *R_t_* is the resistance value at *t* °C, and *R*_0_ is the resistance value at 0 °C. According to IEC751 international standards, the temperature coefficient of the platinum resistance is 0.003851. In a low temperature environment, platinum resistance has better linearity and smaller measurement errors. Furthermore, a platinum resistance wire, with a faster response speed, can be in contact with the external environment.

### 3.2. Property Measurement

**Figure 3.**
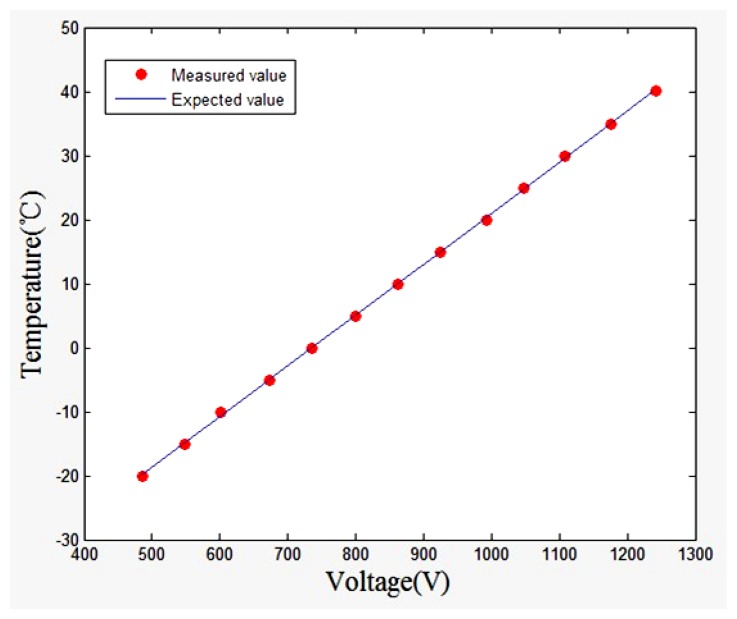
Regression analysis result.

Temperature sensor performance testing was undertaken in an alcohol trough. The temperature was increased from −20 °C to +50 °C in 25 min. The corresponding regression analysis result is shown in Figure 3.

Putting the data into the regression equation, we obtained *R* = 0.9999, *SSE* = 0.9191. The results show that the actual temperature value is very close to the theoretical one. Such a small error reveals a successful estimation of the temperature data.

## 4. Fabrication and Property Measurement of the Temperature Sensor

The sensitive material of the humidity sensor is polyimide. The dielectric constant for the polyimide film with absorbed water can be given by Looyenga's equation [21]:

(13) $$\varepsilon_{s}={\left[\gamma (\varepsilon_{\omega }^{\frac{1}{3}}-\varepsilon_{P}^{\frac{1}{3}})+\varepsilon_{P}^{\frac{1}{3}}\right]}^{3}$$

where *γ* stands for the volume fraction of water in the polyimide film. This sensing capacitance of this type of sensors is given by [28]:

(14) $$C_{s}=\frac{\varepsilon_{0}\varepsilon_{s}A}{d}$$

where *ε*_0_ stands for the vacuum dielectric constant, *d* refers to the distance between the capacitor plates and *A* is the capacitor sensing area.

### 4.1. Fabrication of the Humidity Sensor

**Figure 4.**
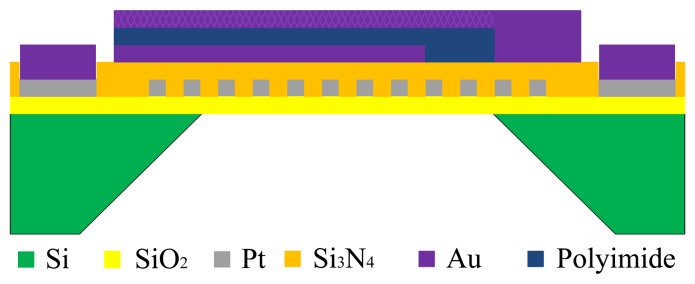
The structure of the humidity sensor.

A sandwich plate capacitive humidity sensor, integrated with a platinum resistance heater, was fabricated using micro electro mechanical system (MEMS) technology. It is composed of a silicon substrate, heating electrode, wet film polyimide sensor and porous gold electrodes. The structure of the humidity sensor is shown in Figure 4.

**Figure 5.**
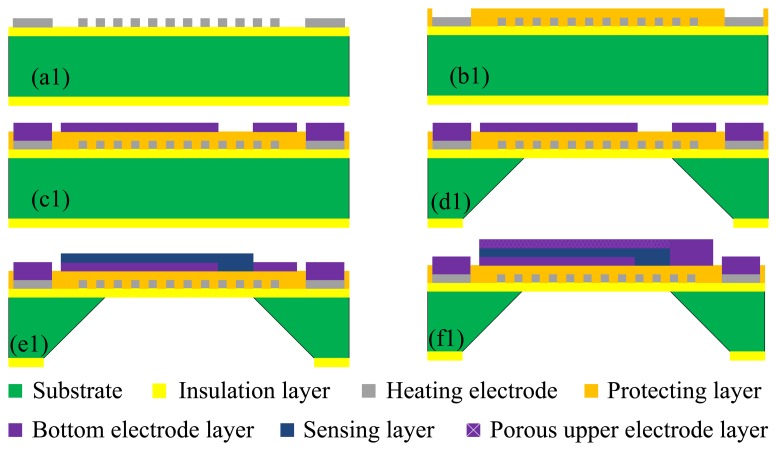
Fabrication flow of the humidity sensor: (**a1**) Snakelike electrode; (**b1**) Dielectric layer; (**c1**) Bottom electrode; (**d1**) Silicon cup; (**e1**) Sensing film; (**f1**) Upper electrode.

**Figure 6.**
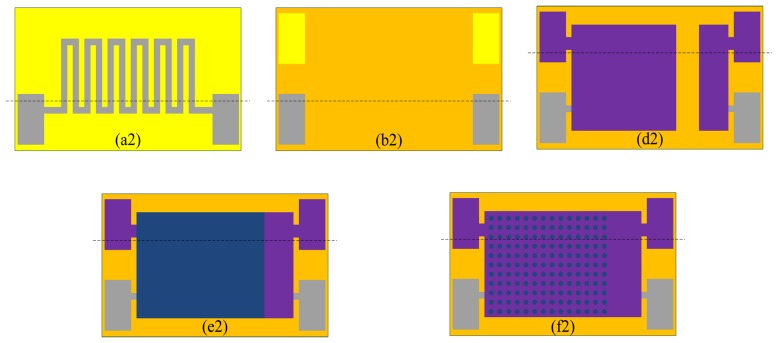
Fabrication flow of planar structure: (**a2**) Snakelike electrode; (**b2**) Dielectric layer; (**d2**) Bottom electrode; (**e2**) Sensing film; (**f2**) Upper electrode.

Figure 5 shows the fabrication flow of the humidity sensor and each fabrication flow of the planar structure is shown in Figure 6.

The Si substrate with a thickness of 150 μm is (100)-oriented. Thermal oxidation technology is used to generate a layer of dense SiO_2_ on the surface of the Si substrate as the first insulation layer. The snakelike heater electrode is fabricated on the insulation layer using photolithographic lift-off technology. The fabrication flow and planar structure are shown in Figure 5a1 and Figure 6a2. Figure 5b1 shows the fabrication flow of dielectric layer using PECVD technology to protect the heater layer. Figure 6b2 shows the planar structure. Figure 5c1 shows the fabrication flow of the Au bottom electrode using photolithographic technology. The silicon cup is fabricated using chemical etching technology with the KOH. Figure 5d1 shows the fabrication flow of silicon cup etching, and Figure 6d2 shows the planar structure. Figure 5e1 shows the fabrication process of sensing film on the Au bottom electrode layer and Figure 5f1 shows the planar structure. Figure 5f1 shows the fabrication process of porous upper Au electrode layer using vacuum evaporation technology, and Figure 6f2 shows the planar structure.

**Figure 7.**
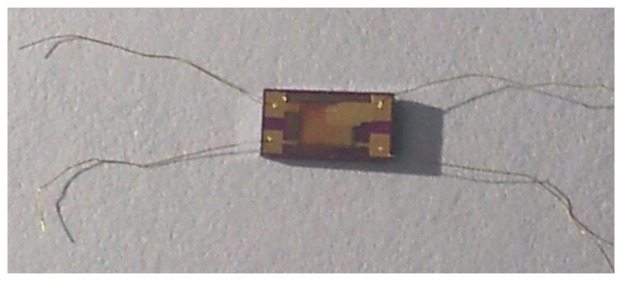
Photograph of the humidity sensor.

A photograph of the humidity sensor is shown in Figure 7. In order to conveniently connect the back-end processing circuit, the gold ball welding method was applied to weld the leads.

### 4.2. Humidity Sensor Property Measurement

**Figure 8.**
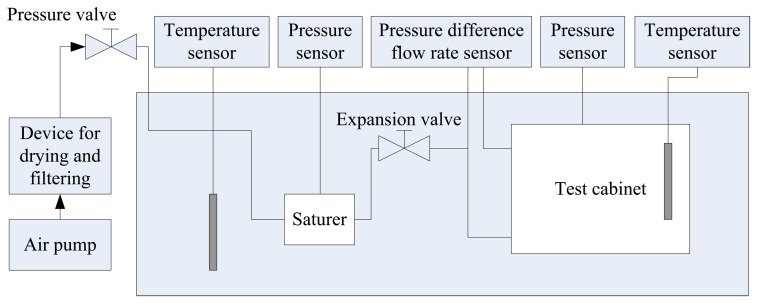
Humidity generator block diagram.

A humidity generator, dew-point instrument and LCR meter were used to measure the performance of the humidity sensor. A dual-pressure method humidity generator is used. It has the advantage that the humidity can be adjusted over a very wide range at different temperatures, and is stable in a short time, so it can be used to study humidity sensor dynamic characteristics. The device block diagram is shown in Figure 8.

**Figure 9.**
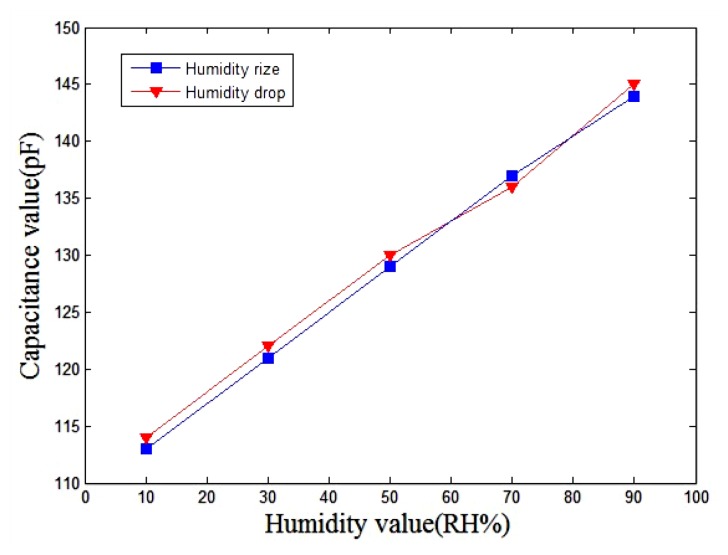
Measured results of the humidity sensor at +20 °C.

Figure 9 shows the measured results of the humidity sensor. In this measurement, the temperature was kept constant at 20 °C and the humidity was increased from 10% RH to 90% RH in 25 min and then dropped to 10% RH at the same rate.

**Figure 10.**
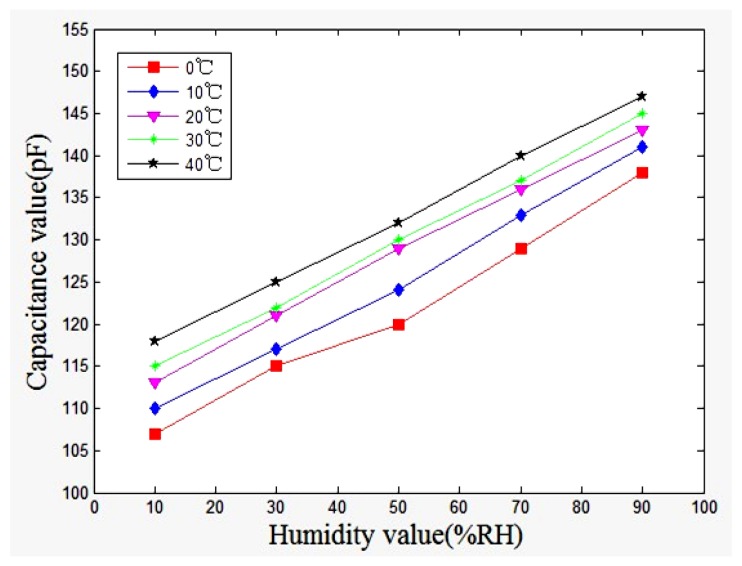
Measured results of the humidity sensor at different temperatures.

The experimental results showed that the humidity sensor has small humidity hysteresis. As shown in Figure 9, the capacitance of the sensor increased from 100 pF to 150 pF, and the sensitivity of the sensor is about 0.375 pF/%RH. The humidity sensor was tested at 0 °C, +10 °C, +20 °C, +30 °C and +40 °C, in order to characterize the influence of temperature by recording the capacitance changes. The sensitivity of the sensor can be obtained by the linear fitting to the data shown in Figure 10. The evaluated results showed that the sensor had a sensitivity of 0.380 pF/%RH at 0 °C and a sensitivity of 0.365 pF/%RH at 40 °C.

**Figure 11.**
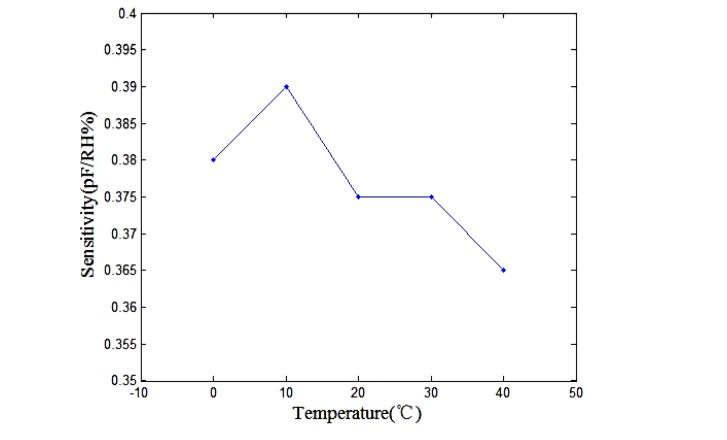
Relation between humidity sensitivity and temperature.

In accordance with the results in Figure 10; the relation between the humidity sensitivity and temperature can be obtained and is shown in Figure 11. The results revealed that the sensor had a high sensitivity under indoor temperature conditions.

In order to obtain the humidity sensor error, a repeatability experiment was carried out from 0 °C to 40 °C, and the humidity sensor error is about 0.3 pF. According to the output characteristics of the sensor, the single shot trigger was used to change the sensor capacitance output into a fixed frequency pulse width output, then, convert the fixed frequency pulse width output into the voltage output according to capacitor charge and dischare. The humidity generator and dew-point instrument were the used to measure the performance of the radiosonde humidity sensor array. The temperature was kept constant around 20 °C and the humidity increases from 10% RH to 90% RH in 50 min. Four humidity sensor outputs are obtained. According to the position of the four sensors placed, the weight of sensors No. 1 and No. 3 is determined to be 1.2, and the weights of sensors No. 2 sensor and No. 4 is 0.8. In accordance with the results in Table 1, *H̄*_1_ and *H̄*_2_ are the two sets of weighted average humidity values, *σ_1_*, *σ_2_* are standard deviations, H_s1_ stands for the fusion humidity based on formula (6). *H̄* stands for the arithmetic average value and *H_S_* stands for the multi-sensor data fusion algorithm's value. One of the experimental data sets is shown in Table 1.

**Figure 12.**
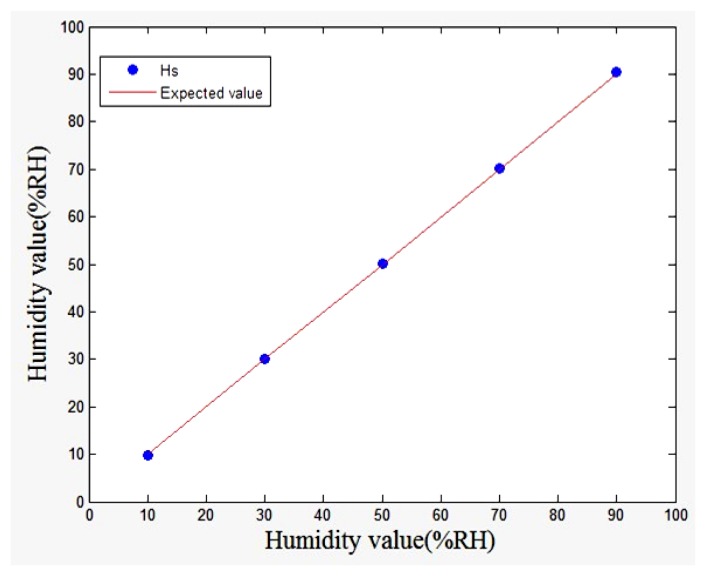
Regression equation result.

Figure 12 shows the humidity data regression equation result, *R* = 0.9998, *SSE* = 0.3528, the result means the actual temperature value and the theoretical temperature value are very close, with small, and successful temperature estimation.

In order to ensure the good performance of the humidity sensor at different temperatures, its surface temperature must be maintained in the range of +20 °C to +30 °C. A high and low temperature testing instrument was used to get the relationship between environment temperature and Pulse-Width-Modulation (PWM). The results were given in Table 2.

Table 3 showed the radiosonde humidity sensor array output values using the heating humidity sensor method and multi-sensor data fusion method. The temperature was increased from −60 °C to +20 °C in 125 min. Humidity increased from 10% RH to 90% RH in 25 min. *H*_−60 °C_, *H*_−40 °C_, *H*_−20 °C_, *H*_0 °C_ and *H*_+20 °C_ stand for the processing humidity values at different temperatures using the multi-sensor data fusion method.

Putting the data into the regression equation, we obtained *R*_−60 °C_ = 0.9986, *SSE*_−60 °C_ = 5.43, *R*_−40 °C_ = 0.9999, *SSE*_−40 °C_ = 4.08, *R*_−20 °C_ = 0.9992, *SSE*_−20 °C_ = 3.125, *R*_0 °C_ = 0.9994, *SSE*_0 °C_ = 2.252, *R*_+20 °C_ = 0.9997, *SSE*_+20 °C_ = 1.202. The result means the actual humidity value and the theoretical humidity value are close. The *SSE* becomes gradually smaller as the environment temperature rises, humidity data estimation is satisfactory, and continuous. The designed humidity with a platinum resistance heater effectively solves the sensor condensation problem.

## 5. Introduction of Pressure Sensor

Considering the requirements of the pressure sensor such as wide operating temperature range, low power consumption, small package and low cost, the MS5540 digital pressure sensor with a resolution of 0.1 hPa and detect range of 10 hPa–1,100 hPa, was chosen and its supply power was 2.2 V–3.6 V. Furthermore, the interior of sensor has a temperature compensation circuit, which makes the pressure sensor still possess a good performance even in low temperature condition.

## 6. Design and Implementation of the Radiosonde System

### 6.1. Hardware Design

**Figure 13.**
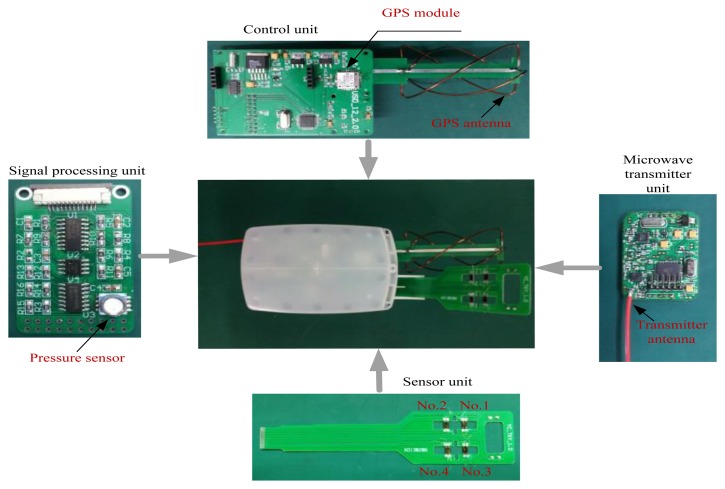
Photographs of the first part system.

**Figure 14.**
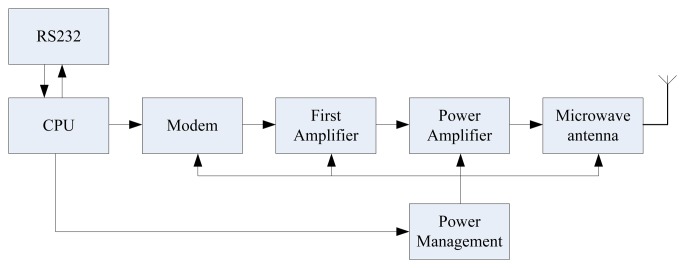
Microwave transmitting block diagram.

The system can be divided into two parts, the first part is data acquisition and transmission, and the second part is data reception and processing. Figure 13 shows photographs of the first part of the system. It consists of a control unit, a sensor unit, a microwave transmitting unit, a GPS unit and a signal processing unit. Figure 14 shows the microwave transmitting block diagram.

**Figure 15.**
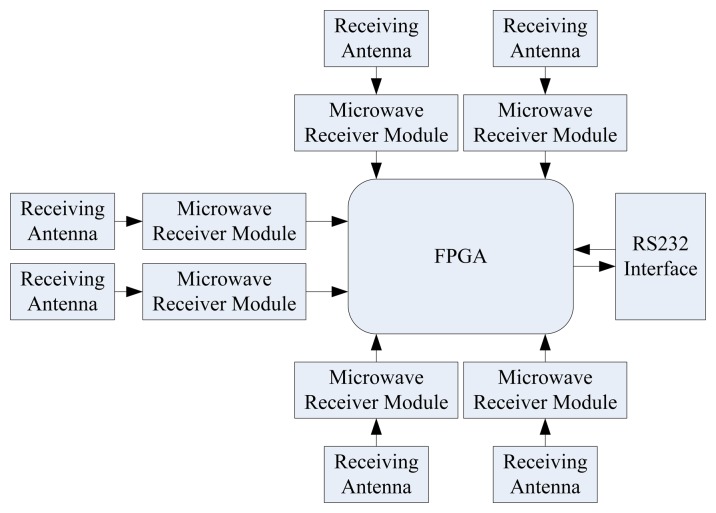
Microwave receiver unit block diagram.

**Figure 16.**
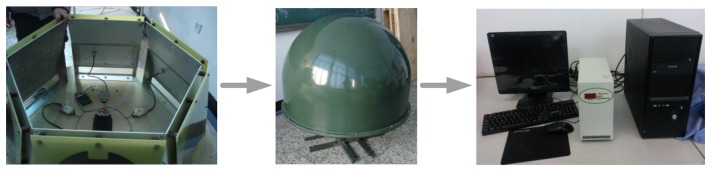
Photograph of the microwave receiver system.

The microwave receiver unit block diagram was shown in Figure 15. Figure 16 shows photographs of the second part of the system, consisting of a microwave receiver unit and computer. The microwave receiver antenna is an omnidirectional antenna, which is constituted by the six receiving antennas. The control chip of microwave receiver unit is FPGA, and its main function is to select the six receiver modules in the strongest signal data as valid data.

### 6.2. Software Design

**Figure 17.**
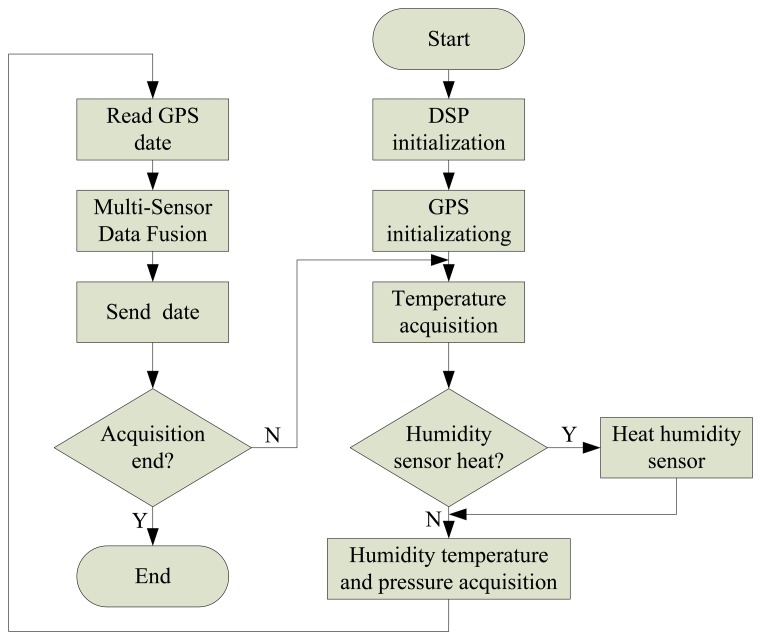
Radiosonde program software flow chart.

According to the hardware architecture of the designed radiosonde, the tasks of the whole system are to achieve high altitude detection of the humidity, temperature and pressure, wireless data transmission and information component display on a PC. Therefore, software development of the system includes two parts—radiosonde software design and PC software design. Figure 17 shows the program flow chart.

The radiosonde software design idea is as follows: first, initialize the DSP and the GPS, collecting the ambient temperature, then, judge whether or not the humidity need to be heated under some temperature conditions, if it needs to be heated, use controlled PWM to regulate the surface of the humidity sensor; secondly, collect the ambient temperature, humidity, pressure, and read the current GPS data; finally, use the multi-sensor fusion method to process humidity data, and use the microwave transmitting unit to send the meteorological data to the ground receiver system.

**Figure 18.**
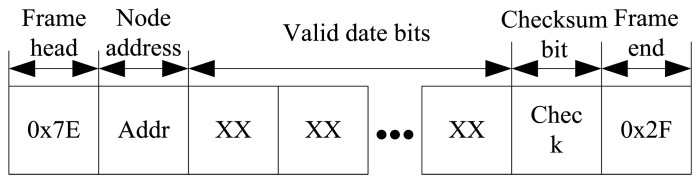
Microwave communication protocol.

In order to reduce the influence of the microwave signal on the received data, and to avoid data loss, the meteorological data is composed of frame head, node address, valid date bits, checksum bit and frame end. The data structure is shown in Figure 18.

**Figure 19.**
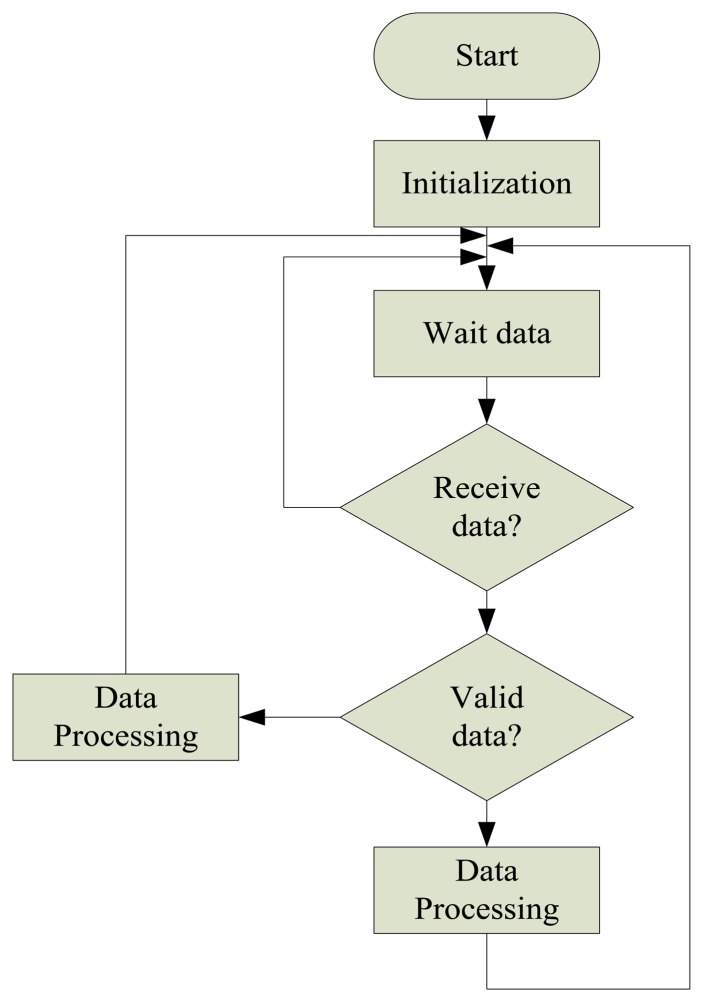
PC program software flow chart.

PC software design idea is as follows: first, initialization procedure, set up the serial number and baud rate, then, wait to receive data until the data is received. Judge validity of the data. If it is valid data, store it and plot the curve, if not, delete it and wait for the next valid data. The data structure is shown in Figure 19.

## 7. Dynamic Experiment Results and Discussion

Dynamic experiments were carried out at one of the meteorological observation stations in the northeast of China, and the meteorological data was collected, including temperature data, humidity data, pressure data, and so on.

### 7.1. Experiment Preparing Process

**Figure 20.**
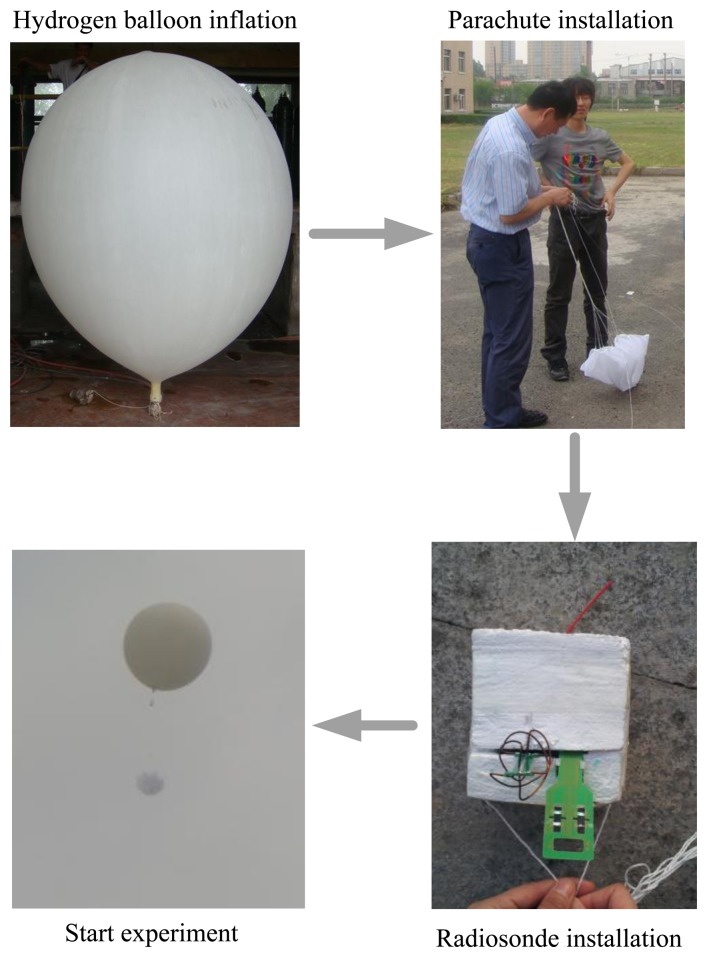
The experiment preparation process.

Figure 20 shows the experiment preparation process. First of all, we prepared the hydrogen balloon, to ensure that the height reached is about 30,000 m. Then we bundle the parachute with rope, the radiosonde is still in the development stage, so the current shell tightness and heat insulation ability are poor, so foam is used to seal the radiosonde. Finally, we fasten the radiosonde to the hydrogen balloon and start the experiment. Experiment duration is about 40 min, and a set of meteorological data were collected. The data is recorded once every 20 s on average.

### 7.2. Dynamic Experimental Results and Discussion

**Figure 21.**
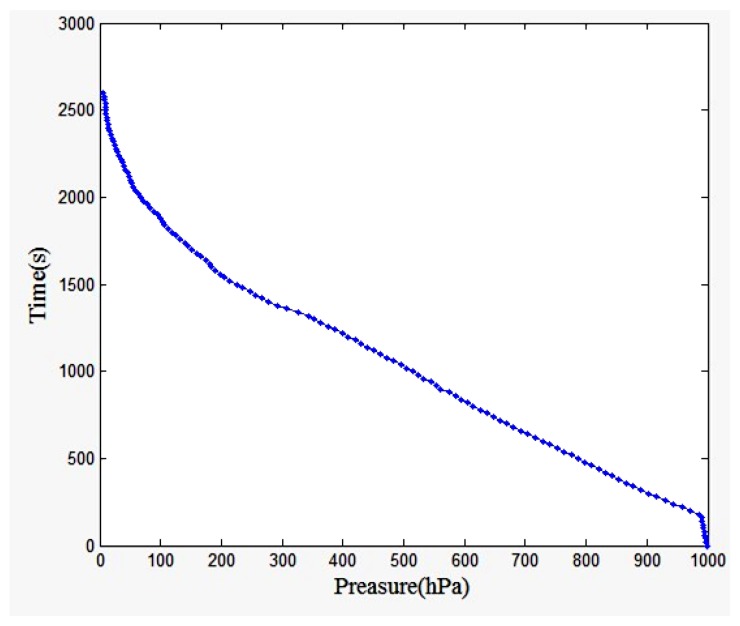
Pressure change over time curve.

**Figure 22.**
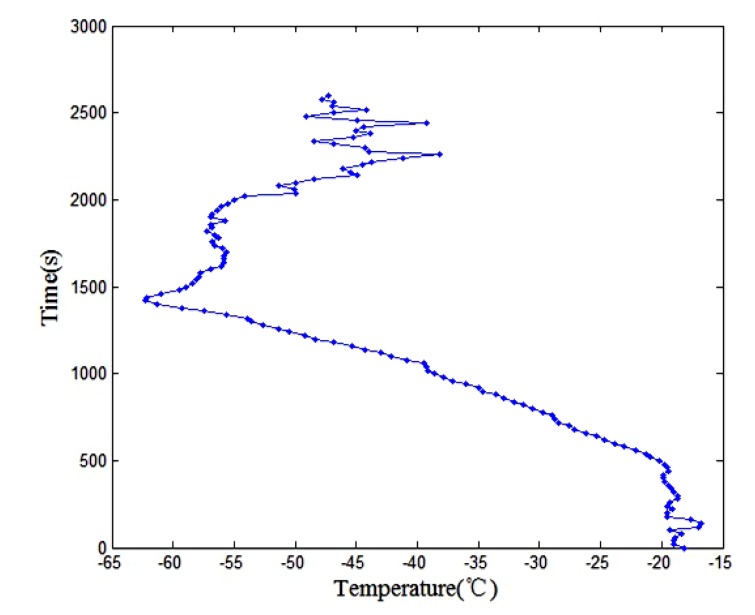
Temperature change over time curve.

Figure 21 showed the pressure change over time curve. The result shows that the pressure is very regular and reveals that the pressure decreases as the height increases. Figure 22 shows the temperature change over time curve. The result shows that from the ground to the top of the troposphere where the altitude is within 15,000 m, when the height increased 1,500 m, the temperature dropped 9 °C. The top of the troposphere altitude is about 15,000 m. Because of the influence of thermal radiation the temperature changes do not follow the basic pattern when the elevation is between 15,000 m and 30,000 m.

**Figure 23.**
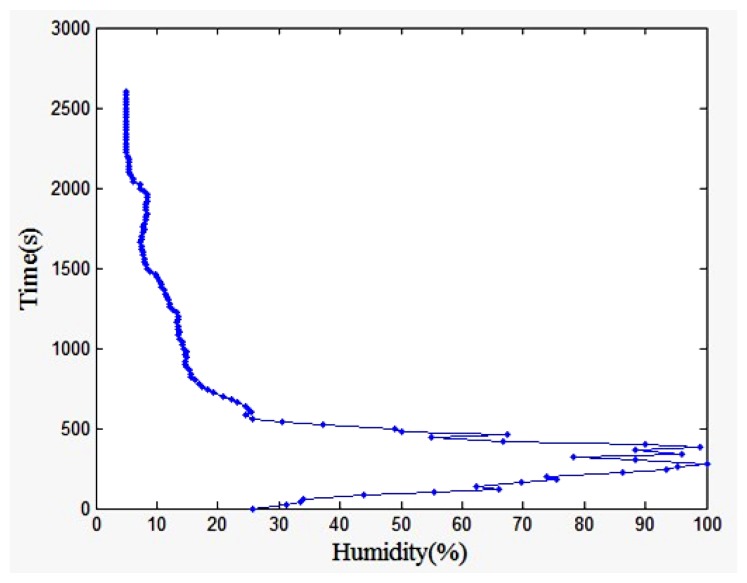
Humidity change over time curve.

Figure 23 showed the humidity change over time curve. The humidity changes abruptly at the altitude of 150 m to 1,000 m. The reason is that in the lower troposphere, under the rubbing action of the atmosphere and surface, the layer exchanges heat and material with the surface occurs, and has turbulent flow characteristics. Furthermore, the humidity sensor is affected by clouds.

The humidity value changes gently at the altitude between 1,000 m and 3,000 m. When the altitude is about 4,000 m to 15,000 m, the layer of water vapor content gradually decreased with increasing altitude, and the humidity gradually reduced. As the altitude continues to increase, the humidity value is essentially constant, because of the water vapor in the air is less and constant.

**Figure 24.**
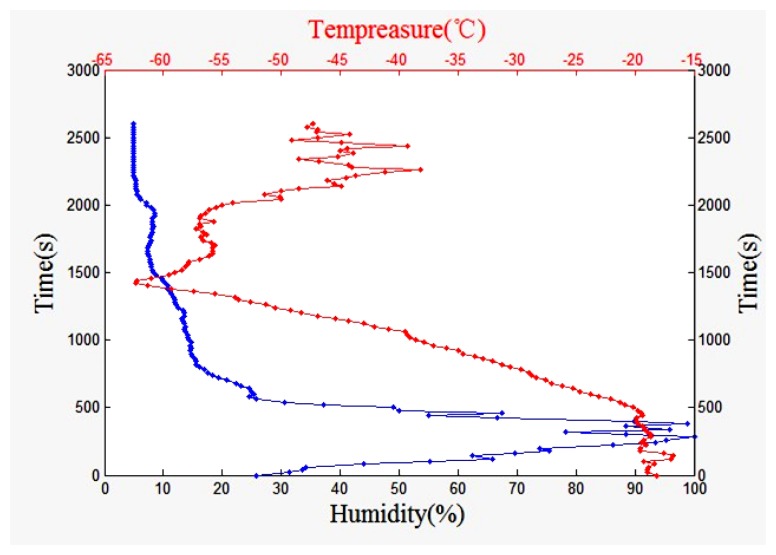
Humidity and temperature change over time curve.

Figure 24 shows the humidity and temperature change over time curve. The humidity sensor has a good performance and the data is continuous under low temperature conditions. The humidity sensor heating method effectively solves the sensor condensation problem.

## 8. Conclusions

A capacitive humidity sensor with a platinum resistance heater was developed using MEMS technology, the capacitance of the sensor increased from 100 pF to 150 pF. Using PWM to control the heating power of humidity sensor, the sensor operating temperature is at about +20–+30 °C, which allows capacitance and humidity to exhibit a good linearity with a sensitivity of about 0.375 pF/%RH. The radiosonde sensor array was made of four humidity sensors to collect aloft atmospheric humidity, and enhance the credibility of the humidity data accuracy.

Since the multi-sensor data fusion technology has the advantages of fault tolerance, complementary real-time, weighted average and batch estimates were used to process the meteorological data. Static experiments show that *R* approached to 1, and the *SSE* was small after the humidity data were processed by the multi-sensor data fusion technology. The actual temperature value was very close to the theoretical one. Such a small error indicates a very successful estimation of the temperature data.

Dynamic experiments showed that the radiosonde worked properly in a high altitude environment with characteristics of high sensitivity and rapid response and can accurately reflect the changes of atmospheric data. The humidity sensor array with a platinum resistance heater can effectively solve the sensor condensation problem. In future work, we will focus on radiosonde data repeatability and reliability, and high altitude comparative experiments with the RS92 unit will be carried out to optimize the hardware and software design.

## Figures and Tables

**Table 1.** Sensor array experimental results using multi-sensor data fusion.

**Set value (%)**	**Sensor output (%)**				***H̄*(%)**	***H̄*_1_(%)**	***H̄*_2_(%)**	***σ*_1_(%)**	***σ****_2_***(%)**	***H****_S_***(%)**

	**1**	**2**	**3**	**4**						
10	11.2	8.2	10.9	8.1	9.60	9.96	9.78	2.153	2.019	9.86
30	31.5	28.4	31.3	28.2	29.85	30.26	30.06	2.235	2.235	30.16
50	51.4	48.6	50.8	48.5	49.83	50.28	49.88	2.019	1.659	50.04
70	71.8	68.5	71.0	68.4	69.93	70.48	69.96	2.380	1.850	70.16
90	92.0	88.1	91.6	88.5	90.05	90.44	90.36	2.812	2.235	90.39

**Table 2.** The relationship between environmental temperature and heating power.

**Temperature Range (°C)**	**PWM Range (%)**	**Sensor Temperature (°C)**
+10∼0	5∼10	28
−10∼−20	10∼15	25
−20∼−30	15∼25	23
−30∼−40	25∼45	22
−40∼−50	45∼70	22
−50∼−60	70∼100	21

**Table 3.** Humidity value regression equation result under the different temperature.

**Set Value**	***H*_−60 °C_**	***H*_−40 °C_**	***H*_−20 °C_**	***H*_0 °C_**	***H*_+20 °C_**
10%	12.2%	12.0%	11.7%	11.5%	11.2
30%	32.1%	31.9%	31.5%	31.2%	31.0%
50%	47.8	48.1%	48.5%	48.9	49.1%
70%	67.2	67.5%	67.3%	67.5%	68.3%
90%	86.9	87.2%	87.6%	88.0%	88.5%
